# Are self-management skills associated with depressive symptoms, quality of life, and healthcare utilisation? A cross-sectional survey among patients with advanced cancer in the Netherlands

**DOI:** 10.1136/bmjopen-2025-108987

**Published:** 2026-02-27

**Authors:** Evi M Bakker, Sophie van Dongen, Erica Witkamp, Leonieke W Kranenburg, Carin C D van der Rijt, Kate Lorig, Natasja Raijmakers, Janneke van Roij, Agnes van der Heide, Judith Rietjens

**Affiliations:** 1 Department of Public Health, Erasmus MC, University Medical Center Rotterdam, Rotterdam, The Netherlands; 2 Research Centre Innovations in Care, Rotterdam University of Applied Sciences, Rotterdam, The Netherlands; 3 Department of Psychiatry, Section of Medical Psychology and Psychotherapy, Erasmus MC, University Medical Center Rotterdam, Rotterdam, The Netherlands; 4 Department of Medical Oncology, Erasmus Medical Center Cancer Institute, Rotterdam, The Netherlands; 5 Self-Management Resource Center, Aptos, California, USA; 6 Netherlands Comprehensive Cancer Organisation (IKNL), Utrecht, The Netherlands; 7 Reinier van Arkel Mental Health Institute, Den Bosch, The Netherlands; 8 Department of Design, Organization, and Strategy, Faculty of Industrial Design Engineering, Delft University of Technology, Delft, The Netherlands

**Keywords:** Cancer, PALLIATIVE CARE, Quality of Life

## Abstract

**Abstract:**

**Objectives:**

Patients with advanced cancer are increasingly encouraged to self-manage the medical, psychosocial, and practical consequences of their illness. However, the impact of self-management skills on patient outcomes and healthcare utilisation remains unclear. Therefore, we examined self-management skills among patients with advanced cancer and their associations with depressive symptoms, quality of life, and formal and informal healthcare utilisation.

**Design and setting:**

We embedded a cross-sectional questionnaire study in a Dutch nationwide prospective observational cohort study (eQuiPe study).

**Participants:**

464 patients with advanced cancer (response rate 42.1%). 50% of the participants were women, and the mean age was 66 years (SD = 10).

**Outcome measures:**

Self-management skills were assessed using three scales of the Health Education Impact Questionnaire: Skill and technique acquisition (STA), Self-monitoring and insight (SMI), and Health services navigation (HSN) (range: 1–4). Multivariate linear and logistic regression analyses were performed to examine associations (adjusting for sociodemographic and medical characteristics) between self-management skills and depressive symptoms (Hospital Anxiety and Depression Scale), quality of life (European Organisation for Research and Treatment of Cancer), and healthcare utilisation in the past month (healthcare organisations and disciplines; hospital admissions; emergency care visits; informal care).

**Results:**

Mean (SD) scores were 3.0 (0.5) for STA, 3.2 (0.4) for SMI, and 3.4 (0.5) for HSN. Higher scores of self-management skills on all three scales were significantly associated with fewer depressive symptoms (STA: β = −2.36, 95% CI −2.98 to −1.69; SMI: β = −2.88, 95% CI −3.64 to −2.09; HSN: β = −2.06, 95% CI −2.76 to −1.37). Patients with higher levels of STA and SMI reported better quality of life (β = 8.54, 95% CI 5.84 to 11.01 and β = 8.41, 95% CI 4.75 to 11.99, respectively). Regarding healthcare utilisation, only HSN showed a significant association, with higher scores being associated with increased nurse contact (β = 1.65, 95% CI 1.09 to 2.56).

**Conclusions:**

Greater self-management skills were associated with fewer depressive symptoms and improved quality of life in patients with advanced cancer. However, self-management skills were hardly associated with healthcare utilisation.

**Trial registration number:**

Netherlands Trial Register; NTR6584.

**STRENGTHS AND LIMITATIONS OF THIS STUDY**

- This observational study assesses associations between patients’ disease-oriented self-management and well-being across various advanced cancer types.
- The response rate of this study was high.
- The cross-sectional design limits the causal interpretation of the associations observed.
- The operationalisation of self-management was limited to disease-oriented self-management skills.

## Background

 The incidence of advanced cancer has increased in the past decades.^1^ Concurrently, patients are living longer with advanced cancer and its consequences.^2^ Patients spend most of their time outside of formal healthcare settings and they have to take an active role in managing their health and well-being themselves.^3^ Self-management of health conditions includes strategies and activities to manage the physical, psychosocial, existential, and medical consequences of living with an illness.^3^ Patients are, for example, expected to self-monitor their symptoms and self-administer medications.^4^ This requires insights into their condition and skills to cope with their health problems. Patients are also expected to navigate healthcare services, including collaborating with healthcare professionals and coordinating medical services.^4^ However, patients with advanced cancer have stated that they experience self-management as strenuous, especially if they are confronted with increasingly debilitating symptoms.^5^ Furthermore, the healthcare system they must navigate through is complex, as are the decision-making processes they engage in, which often comprise difficult trade-offs between survival and quality of life.^3^,^7^

Self-management has predominantly been studied for patients with chronic diseases, including asthma and diabetes.^3 8^ Research on self-management of patients with chronic diseases showed that better self-management can result in maintained healthy lifestyle habits, better quality of life, and fewer healthcare visits and hospitalisations.^8^,^14^ However, results obtained for patients with chronic, often non-lethal, diseases cannot simply be generalised to patients with advanced cancer.^8^ The anticipation of rapid health decline and death is expected to influence self-management strategies and experiences because of ‘changes in values, priorities, interests and social interactions’.^4^ Patients could, for example, weigh the trade-off between quality and quantity that treatments could yield differently as the end of life approaches.^3 4 15^ However, population-based studies among patients with advanced cancer on self-management skills and their association with well-being and care utilisation are scarce. Therefore, this study aimed to assess self-management skills and their association with depressive symptoms, quality of life, and formal and informal care utilisation among patients with advanced cancer.

## Methods

### Study design and population

This cross-sectional questionnaire study is nested in the eQuiPe study, a prospective longitudinal observational cohort study on the experienced quality of care and quality of life of Dutch patients with advanced cancer and their relatives.^16^ In the current study, we report only self-reported patient data. All patients with a solid metastasised tumour (stage IV) were eligible for inclusion. To reduce the overrepresentation of patients with a relatively good prognosis, patients diagnosed with breast and prostate cancer were only eligible if they had metastases in multiple organ systems or castrate-resistant cancer, respectively. Additionally, participants had to be aged >18 years, be able to complete a self-report questionnaire in Dutch, understand the objective of the study, have no history of severe psychiatric illness, and have signed the informed consent form.

### Study procedure

From November 2017 to January 2020, patients in 1 of 40 participating hospitals in the Netherlands were invited to the study by their treating physician. Patients could also participate through self-referral, by leaving a contact request on a Dutch internet platform for patients with cancer (www.kanker.nl) on which the study was advertised. After giving written informed consent for the eQuiPe study, patients were asked to complete a questionnaire every 3 months until death. Participants received questionnaires on paper or online via the Patient Reported Outcomes Following Initial Treatment and Long-term Evaluation of Survivorship registry.^17^ A reminder letter and paper version of the questionnaire were sent to patients who did not return a questionnaire within 2 weeks. If the questionnaire was not completed within 2 weeks after this reminder, the patient was contacted by phone.

While the eQuiPe study was ongoing, we added questions regarding self-management skills and depressive symptoms to the first follow-up questionnaire. In the current study, we combined cross-sectional data from patients who completed both the baseline questionnaire and the measures of self-management skills and depressive symptoms of the first follow-up questionnaire. Detailed information on the cohort study has been published elsewhere.^16^

### Patient and public involvement

To improve the final questionnaire, both the original eQuiPe questionnaire and the additional items as part of this study were pilot-tested among patients with advanced cancer and their relatives (n=38). Participants provided feedback on completion time, appropriateness, understandability, and response burden. As we have no contact details of participants of the eQuiPe study, the study results will not be directly disseminated to the participants.

### Measures

#### Self-management

Self-management was assessed at the first follow-up time point, with the Health Education Impact Questionnaire (heiQ), a validated self-report questionnaire for evaluating self-management skills across various health conditions.^18^ Statements assess self-management knowledge, self-efficacy, and behaviour. It has demonstrated good psychometric properties and has been validated among patients with cancer.^18^ A Dutch version has been culturally adapted and validated.^19^ The heiQ comprises 40 items across eight self-management scales. We selected three scales that we considered most related to disease-oriented self-management skills and therefore most relevant for the purpose of our study: Skill and technique acquisition (STA), Self-monitoring and insight (SMI), and Health services navigation (HSN). Responses are marked on a 4-point Likert scale (‘strongly disagree’ to ‘strongly agree’). Mean scores (range 1–4) were calculated per scale. Higher scores indicate higher levels of: skills in symptom relief and techniques to manage one’s health (STA); skills in self-monitoring, setting reasonable limits or targets, and insight into living with a health problem (SMI); and confidence in one’s ability to communicate with healthcare professionals and understanding ways to access healthcare to get one’s needs met (SMI).

#### Depressive symptoms

Depressive symptoms were assessed at the first follow-up time point with the seven-item subscale of the widely validated Hospital Anxiety and Depression Scale (HADS) questionnaire, which asks patients to report the frequency or severity of specific depression-related mood states during the past week on a 4-point ordinal response scale (0–3).^20^ After recoding the negatively formulated items, a sum score was calculated (range 0–21). Higher scores indicate greater levels of depressive symptoms. Scores ≥11 reflect severe depressive symptoms.

#### Quality of life

Quality of life was measured at baseline by the 30-item version of the European Organisation for Research and Treatment of Cancer Quality of Life Questionnaire (EORTC QLQ-C30), comprising five functional subscales (physical, role, cognitive, emotional, and social), a global quality of life subscale, three symptom subscales (fatigue, pain, and nausea and vomiting), and six single items (appetite loss, diarrhoea, dyspnoea, constipation, insomnia, and financial impact).^21^ Patients are asked to report on a 4-point Likert scale to what extent they experienced problems during the past week, except for the global quality of life subscale, which asks a seven-point response rating for overall health and quality of life during the past week. According to the EORTC guidelines, subscale and single-item scores were linearly transformed to a score between 0 and 100. For the functioning and the global quality of life subscales, higher scores reflect better functioning and health. For the symptom subscales, higher scores indicate more symptom burden. In line with Giesinger *et al.*, we composed a single summary score as the mean of 13 of the combined subscales and item scores (excluding global quality of life and financial impact; 27 of 30 items).^22^ The summary score was only calculated when all thirteen scale and item scores were available.^22^ A higher score indicates a better quality of life.

#### Healthcare service and informal care utilisation

Healthcare service utilisation in the past month and informal care utilisation in the past week were assessed at baseline with items developed based on the Dutch Quality Framework of Palliative Care.^23^ Patients were asked whether they had been in any type of contact with various healthcare professionals for their care, and if so, how often. Further, patients were asked if they had used hospital and home care. Hospital admissions were examined by asking if patients had stayed overnight. Emergency care visits were examined by asking patients whether they had visited the out-of-hours general practitioner service or hospital emergency department. Informal care utilisation was estimated by asking patients if they had received care or assistance from their relatives (eg, partner, family, friend, neighbour and colleague) and if so, how many hours.

#### Sociodemographic and clinical characteristics

Sociodemographic and clinical characteristics of patients were collected at baseline, including their age, gender, ethnicity (‘what is your ethnicity’), educational level, marital status, having children, life stance (‘having a particular philosophy of life’), self-reported comorbidity Self-administered Comorbidity Questionnaire,^24^ and self-estimated life expectancy. Education was categorised into three categories (low-medium-high) according to the International Standard Classification of Education guidelines.^25^ Clinical data were obtained through linkage with the Netherlands Cancer Registry and included primary cancer type, stage, and time of diagnosis.

### Data analysis

Descriptive statistics were used to describe background characteristics, self-management skills, depressive symptoms, quality of life and care utilisation. Bivariate linear and logistic regression analyses were performed to investigate associations between the three self-management skills scales and background characteristics, depressive symptoms, quality of life and care utilisation. Multivariate linear and logistic regression analyses were performed to assess associations between the three self-management skills scales (independent variable) and depressive symptoms, quality of life and care utilisation. The multivariate regression analyses were adjusted for background characteristics that were borderline significantly (p<0.2) associated with self-management skills scales in bivariate analyses. Within these regression analyses, bootstrapping (1000×) was applied to overcome the problem of skewed distributions of the dependent variables. No imputation of missing values was performed. P values <0.05 were considered statistically significant. We analysed data with IBM SPSS V.25.

## Results

### Study population

**Figure 1:**
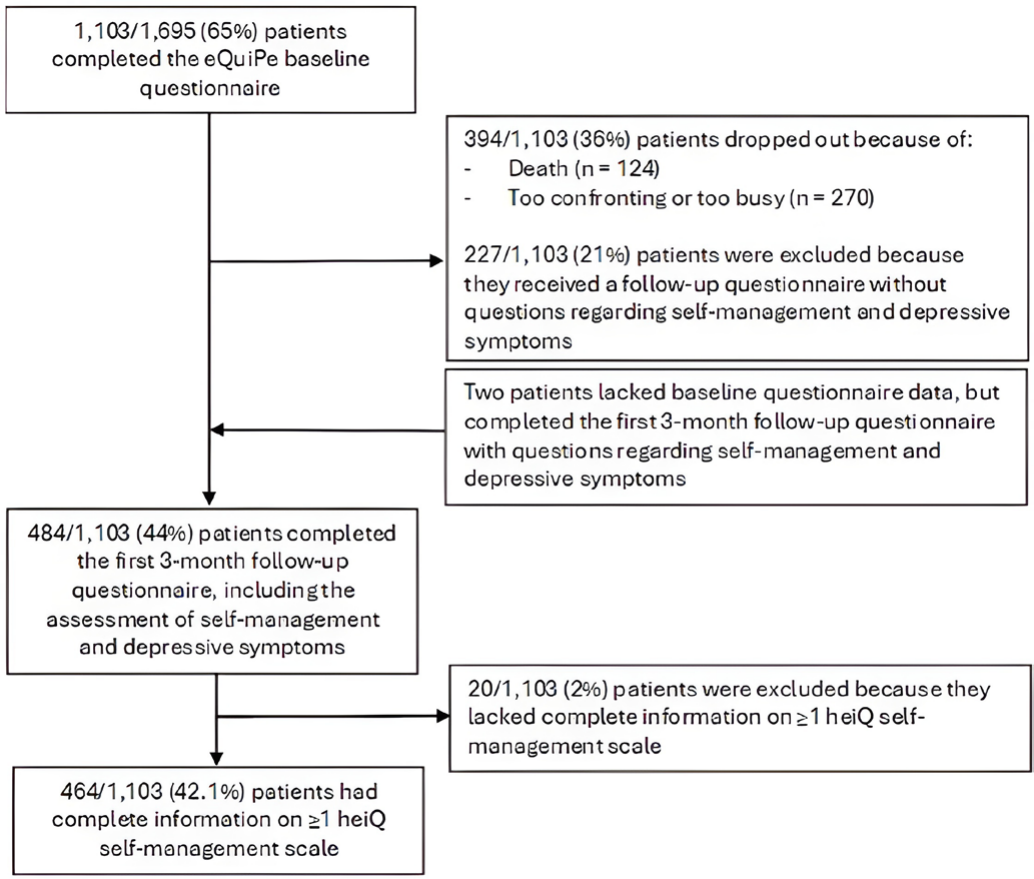
Flowchart of the selection process of the study population.

A total of 464 patients were included in the analysis (figure 1). 1103 patients completed the eQuiPe baseline questionnaire. 484 patients participated in the first 3-month follow-up assessment, including the additional questions regarding self-management skills and depressive symptoms. 464 patients (42.1%) completed at least one full heiQ scale.

Participants’ mean (SD) age at baseline was 65.7 (10.1) years, and half (50.4%) were female (table 1). The majority reported having a Dutch background (96.5%), a medium (41.2%) or high (29.2%) educational level, living together with their partner (76.5%), having one or more children (83.0%), and a specific conviction about life and/or religion (64.3%). Patients were diagnosed with different primary tumour types: lung (25.1%), breast (18.1%), colorectal (16.8%), prostate cancer (15.5%), or other (24.5%). Most patients (88.7%) received medical cancer treatment in the past 3 months. Half the patients were diagnosed 1–5 years prior to the baseline questionnaire (50.7%). Almost half of the patients were able or willing to estimate their life expectancy. 11.6% of the patients estimated this as <1 year, and 34.2% as >1 year.

**Table 1:** Sociodemographic and medical characteristics, well-being, and formal and informal care use of patients with advanced cancer (n=464)

Sample characteristic	Sample description
Sociodemographic characteristics	n (%)
Age (years), mean (SD); range	65.7 (10.1); 29–93
Gender	
Male	230 (49.6)
Female	234 (50.4)
Dutch ethnicity	444 (96.5)
Educational level	
Low	136 (29.6)
Medium	189 (41.2)
High	134 (29.2)
Marital status–living together	354 (76.5)
Children	
No	78 (17.0)
Yes, not living at home	318 (69.4)
Yes, and living at home	62 (13.5)
Having a philosophy about life or religious affiliation	296 (64.3)

Clinical characteristics	n (%)
Primary tumour site	
Lung	115 (25.1)
Colorectal	77 (16.8)
Breast	83 (18.1)
Prostate	71 (15.5)
Other	112 (24.5)
Time since primary tumour diagnosis	
<1 year	104 (22.7)
1–5 years	232 (50.7)
>5 years	122 (26.6)
Life expectancy in years according to patient	
<1 year	52 (11.6)
>1 year	153 (34.2)
Other (I don’t know/I haven’t been told/I don’t want to know)	242 (54.1)
Number of comorbid conditions	
0	148 (32.4)
1	154 (33.7)
>1	155 (33.9)
Cancer treatment in past 3 months (more than one option possible)	401 (88.7)
No	51 (11.3)
Chemotherapy	222 (49.1)
Radiotherapy	48 (10.6)
Surgery	14 (3.1)
Immunotherapy (eg, targeted therapy)	115 (25.4)
Other	103 (22.8)

Well-being	Median (25th–75th percentile)
HADS Depressive symptoms*	3.0 (1.0–6.0)
EORTC QLQ-C30 Quality of life summary score†	82.1 (70.7–90.6)
EORTC QLQ-C30 Function scales†	
Emotional	83.3 (66.7–91.7)
Social	83.3 (66.7–100.0)
Global	66.7 (58.3–83.3)
Role	66.7 (50.0–100.0)
Cognitive	83.3 (66.7–100.0)
Physical	77.8 (60.0–86.7)

Healthcare service use	**n (%)**
Had contact with healthcare discipline at least once in the past month (more than one option possible)	
Medical care‡	385 (85.0)
Nursing care§	289 (63.8)
Care from other professionals¶	163 (36.3)
Received care from healthcare organisation at least once in the past month (more than one option possible)	
Hospital	329 (78.5)
Homecare	71 (16.9)
At least one hospital admission in the past month	42 (9.6)
At least one emergency care visit in the past month	29 (6.6)

Use of informal care in the past week	**n (%**)
Yes**	263 (57.9)
If yes: number of hours per week, median (25th–75th percentile)	8.0 (3.0–20.0)

Missing range was 2–15, with exception for life expectancy (n=17), quality of life (n=20), healthcare organisation(s) involved (n=45), hospital admission (n=28), and emergency care visits (n=26).

* HADS (range: 0–21; higher scores indicate more depressive symptoms). Measured at the first 3-month follow-up time point.

† EORTC QLQ-C30 (range: 0–100; higher scores indicate better quality of life or better function).

‡ Medical care: medical specialist, general practitioner, radiological assistant.

§ Nursing care: nurse specialist, oncology nurse, homecare nurse.

¶ Care from other professionals: psychologist, sexologist, social worker, chaplain, palliative care team, physiotherapist, occupational therapist, dietitian, complementary and alternative care worker.

** Informal caregiver(s): partner, child(ren), family, neighbours, friends, colleagues, other.

EORTC QLQ-C30, European Organisation for Research and Treatment for Cancer Quality of Life Questionnaire; HADS, Hospital Anxiety and Depression Scale.

### Self-management skills

The mean (SD) scale score for Skill and technique acquisition was 3.00 (0.52, scale range 1–4) (table 2). The mean (SD) scale score for Self-monitoring and insight was 3.15 (0.42, scale range 1–4). The mean (SD) scale score for Health services navigation was 3.36 (0.47, scale range 1–4).

**Table 2:** Self-management scores on three domains of the Health Education Impact Questionnaire (heiQ) among patients with advanced cancer (n=464)

Self-management: heiQ*	n	Mean (SD)
Skill and technique acquisition (STA)	455	3.00 (0.52)
I have effective ways to prevent my symptoms (eg, pain, discomfort, stress) from limiting what I can do in my life.	455	2.89 (0.73)
I have a very good idea of how to manage my health problems.	455	3.09 (0.60)
When I have symptoms, I have the skills that help me cope.	455	3.01 (0.59)
I am very good at using aids and devices to make my life easier.	455	3.00 (0.69)

Self-monitoring and insight (SMI)	451	3.15 (0.42)
As well as seeing my doctor, I regularly monitor changes in my health.	463	2.94 (0.75)
I know what things can trigger my health problems and make them worse.	460	3.07 (0.77)
I have a very good understanding of when and why I am supposed to take my medication.	459	3.55 (0.52)
When I have health problems I have a clear understanding of what I need to do to control them.	463	2.95 (0.66)
I carefully watch my health and do what is necessary to keep as healthy as possible.	464	2.24 (0.52)
With my health in mind, I have realistic expectations of what I can and cannot do.	462	3.14 (0.61)

Health services navigation (HSN)	458	3.36 (0.47)
I have very positive relationships with my healthcare professionals.	458	3.32 (0.55)
I communicate very confidently with my doctor about my healthcare needs.	458	3.34 (0.59)
I confidently give healthcare professionals the information they need to help me.	458	3.44 (0.56)
I get my needs met from available healthcare resources (eg, doctors, hospitals, and community services).	458	3.34 (0.56)
I work in a team with my doctors and other healthcare professionals.	458	3.37 (0.56)

Missings: STA (n=10), SMI (n=14) and HSN (n=6).

Measured at the first 3-month follow-up time point.

* HeiQ item and scale scores range from 1 (‘strongly disagree’) to 4 (‘strongly agree’). Higher scores indicate higher levels of self-management skills.

### Depressive symptoms, quality of life, and healthcare service and informal care utilisation

The median (IQR) depressive symptom score was 3.0 (1.0–6.0, scale range 0–21) (table 1). 6% of participants reported severe depressive symptoms (not tabulated). The median (IQR) quality of life summary score was 82.1 (70.7–90.6, scale range 0–100). Most patients stated that they had received medical care from a general practitioner and/or medical specialist (85.0%) and/or nursing care (63.8%) in the previous month. 36.3% reported receiving care from other professionals in the past month, for example, from a psychologist, physiotherapist, or dietitian. 78.5% of patients received hospital care in the past month and 16.9% received home care in the past month. 9.6% of participants indicated having been admitted to the hospital and 6.6% reported emergency care visits in the past month. Almost 60% of the patients indicated they received informal care from a relative, which in most cases concerned their partner (46.3%) and the median (IQR) number of hours per week was 8.0 (3.0–20.0).

### Associations between self-management skills and depressive symptoms and quality of life

Higher self-management scores were associated with lower depressive symptom scores. This was true for all three self-management scales, also after adjusting for background characteristics (Skill and technique acquisition: β=−2.36, 95% CI −2.98 to −1.69; Self-monitoring and insight: β=−2.88, 95% CI −3.64 to −2.09; Health services navigation: β=−2.06, 95% CI −2.76 to −1.37) (model 2 in table 3). Bivariate linear regression analyses showed that higher self-management scores for all three scales were associated with a better quality of life (model 1 in table 3). After adjusting for background characteristics, this remained true for the scales Skill and technique acquisition (β=8.54, 95% CI 5.84 to 11.01) and Self-monitoring and insight (β=8.41, 95% CI 4.75 to 11.99) (model 2 in table 3).

**Table 3:** Bootstrapped (1000×) linear regression for the associations between heiQ self-management subscales and depressive symptoms and quality of life among patients with advanced cancer

heiQ subscale	Depressive symptoms (HADS)*			Quality of life summary score (QLQ-C30)		
STA†	β‡	**95% CI§**	P value	β‡	**95% CI§**	P value
Model 1 (bivariate)	**−2.45**	**−3.07 to −1.81**	**<0.01**	**9.35**	**6.59 to 11.93**	**<0.01**
Model 2 (adjusted)	**−2.36**	**−2.98 to −1.69**	**<0.01**	**8.54**	**5.84 to 11.01**	**<0.01**

SMI¶	β‡	95% CI§	P value	β‡	95% CI§	P value
Model 1 (bivariate)	**−2.96**	**−3.74 to −2.19**	**<0.01**	**8.64**	**4.87 to 12.29**	**<0.01**
Model 2 (adjusted)	**−2.88**	**−3.64 to −2.09**	**<0.01**	**8.41**	**4.75 to 11.99**	**<0.01**

HSN**	β‡	95% CI§	P value	β‡	95% CI§	P value
Model 1 (bivariate)	**−2.08**	**−2.76 to −1.39**	**<0.01**	**3.23**	**0.16 to 6.36**	**0.045**
Model 2 (adjusted)	**−2.06**	**−2.76 to −1.37**	**<0.01**	2.96	−0.17 to 5.89	0.06

* Direction of the association is reversed because higher HADS scores indicate more depressive symptoms.

† Range 1-4 ;Model 1=bivariate; Model 2=adjusted for educational level, time since diagnosis, life expectancy, and comorbidities.

‡ Statistically significant results (p<0.05) are in bold.

§ Bootstrap for coefficients (1000×).

¶ Range 1-4; Model 1=bivariate; Model 2=adjusted for educational level.

** Range 1-4; Model 1=bivariate; Model 2=adjusted for cultural background and comorbidities.

HADS, Hospital Anxiety and Depression Scale; heiQ, Health Education Impact Questionnaire; HSN, Health services navigation; QLQ-C30, Quality of Life Questionnaire Core 30; SMI, Self-monitoring and insight; STA, Skill and technique acquisition; β, regression coefficient.

### Associations between self-management skills and healthcare service and informal care utilisation

In general, higher self-management scores were not associated with healthcare service and informal care utilisation. In bivariate logistic regression analyses, higher levels of Skill and technique acquisition were associated with not having an informal caregiver. Furthermore, higher levels of Health services navigation were associated with more use of nursing care in the past month (model 1 in table 4). After adjustment for background characteristics, the results were robust only for the association between Health services navigation and the use of nursing care (β=1.65, 95% CI 1.09 to 2.56) (model 2 in table 4). No associations were found between self-management skills and the involvement of medical care or care from other professionals, nor the involvement of healthcare organisations, nor for hospital admissions or emergency care visits in the past month.

**Table 4:** Bootstrapped (1000×) logistic regression for the associations between heiQ self-management subscales and care use among patients with advanced cancer

heiQ subscale	Involvement in the past month of									Involvement in the past month of						Hospital admissions in the past month			Emergency care visits in the past month			Involvement of informal caregiver in the past week		
	Medical care			Nursing care			Care from other professionals			Hospital care			Home care											
STA*	β†	95% CI‡	P value	β†	95% CI‡	P value	β†	95% CI‡	P value	β†	95% CI‡	P value	β†	95% CI‡	P value	β†	95% CI‡	P value	β†	**95% CI‡**	P value	β†	**95% CI‡**	P value
Model 1	0.94	0.56 to 1.57	0.83	0.87	0.59 to 1.28	0.49	0.85	0.57 to 1.28	0.41	0.79	0.48 to 1.28	0.36	0.82	0.49 to 1.38	0.50	0.97	0.51 to 1.84	0.94	1.10	0.58 to 2.01	0.75	**0.66**	**0.44 to 0.99**	**0.04**
Model 2	0.86	0.44 to 1.61	0.66	0.88	0.57 to 1.29	0.53	0.87	0.57 to 1.30	0.49	0.76	0.44 to 1.26	0.31	0.89	0.50 to 1.56	0.70	1.04	0.52 to 2.05	0.91	1.21	0.60 to 2.40	0.58	0.71	0.45 to 1.11	0.10

SMI§	β†	95% CI‡	P value	β†	95% CI‡	P value	β†	95% CI‡	P value	β†	95% CI‡	P value	β†	95% CI‡	P value	β†	95% CI‡	P value	β†	**95% CI‡**	P value	β†	**95% CI‡**	P value
Model 1	1.04	0.55 to 1.98	0.89	1.16	0.71 to 1.90	0.53	0.81	0.49 to 1.32	0.40	0.62	0.32 to 1.12	0.11	0.73	0.36 to 1.43	0.34	1.08	0.46 to 2.34	0.87	1.95	0.67 to 6.23	0.18	1.14	0.72 to 1.84	0.60
Model 2	0.91	0.45 to 1.88	0.78	1.17	0.73 to 1.90	0.49	0.76	0.47 to 1.23	0.27	0.58	0.29 to 1.07	0.08	0.84	0.39 to 1.75	0.63	1.07	0.44 to 2.31	0.86	2.19	0.72 to 7.86	0.12	1.19	0.76 to 1.98	0.51

HSN¶	β†	95% CI‡	P value	β†	95% CI‡	P value	β†	95% CI‡	P value	β†	95% CI‡	P value	β†	95% CI‡	P value	β†	95% CI‡	P value	β†	**95% CI‡**	P value	β†	**95% CI‡**	P value
Model 1	1.13	0.64 to 1.98	0.72	**1.59**	**1.06 to 2.45**	**0.03**	1.01	0.64 to 1.56	0.97	0.79	0.45 to 1.36	0.39	1.29	0.73 to 2.22	0.41	0.66	0.28 to 1.33	0.30	1.00	0.43 to 2.37	1.00	0.90	0.59 to 1.36	0.62
Model 2	1.16	0.60 to 2.12	0.66	**1.65**	**1.09 to 2.56**	**0.02**	1.05	0.67 to 1.64	0.84	0.77	0.43 to 1.32	0.34	1.33	0.74 to 2.32	0.36	0.69	0.28 to 1.46	0.37	1.04	0.43 to 2.46	0.93	0.94	0.61 to 1.42	0.76

* Range 1-4; Model 1=bivariate; Model 2=adjusted for educational level, time since diagnosis, life expectancy, and comorbidities.

† Statistically significant results (p<0.05) are in bold.

‡ Bootstrap for coefficients (1000×).

§ Range 1–4; Model 1=bivariate; Model 2=adjusted for educational level.

¶ Range 1–4; Model 1=bivariate; Model 2=adjusted for cultural background and comorbidities.

heiQ, Health Education Impact Questionnaire; HSN, Health services navigation; SMI, Self-monitoring and insight; STA, Skill and technique acquisition.

## Discussion

### Summary of findings

Our cross-sectional questionnaire study among 464 patients with various types of advanced cancer demonstrated relatively high levels of self-management skills. Patients with higher self-management scores generally reported fewer depressive symptoms and a better quality of life. We found no clear associations between self-management skills and healthcare services or informal care utilisation.

### Strengths and limitations

This study has several strengths. It is among the first large observational studies to demonstrate positive associations between disease-oriented self-management and well-being across advanced cancer types. Second, response rates were relatively high.^16^ However, the cross-sectional design limits causal inference, underscoring the need for longitudinal research. Potential biases may have influenced the results. Recruitment by treating physicians may have favoured participation of patientsx’ with positive healthcare interactions, potentially overrepresenting those with higher levels of health service navigation skills. Furthermore, our population lacked ethnic diversity, most participants had medium or high educational attainment, and those with severe psychiatric illness were excluded from the eQuiPe study. The level of depressive symptoms in our study population could be considered better than expected as it was comparable with normative data, and quality of life resembled that of patients with localised cancer.^22 26 27^ Last, our operationalisation of self-management was limited to disease-oriented self-management skills. Broader conceptualisations of self-management, for instance, also include emotional self-management, focusing on handling the psychological process of adaptation to being ill.^28^ Besides, the items focus on the individual, omitting the care system or social network, and the possible dyadic and reciprocal nature of support between patients and informal caregivers.^29^

### Levels of self-management skills

The mean heiQ self-management skill scores were close to 3 on a 1–4 scale and are considered relatively high.^30^ This indicates that our population of patients with advanced cancer has relatively well-developed skills in ‘symptom relief and techniques to manage one’s health’; ‘self-monitoring, setting reasonable limits or targets, and insight into living with a health problem’; and ‘were confident in their ability to communicate with healthcare professionals and had a good understanding of ways to access healthcare to get their needs met’.^18^ Our results are comparable with studies among patients living in Canada with various types and stages of cancer, including advanced cancer, that previously demonstrated that patients appear to deploy proficient self-management skills in coping with their condition.^30 31^ These self-management skills found among patients with cancer are higher compared with those of patients with mild chronic obstructive pulmonary disease (COPD).^32^ This difference in self-management skills could be attributed to differences in educational levels. In our, as well as the previous studies, the levels of educational attainment in the population of patients with cancer were higher compared with the educational levels in the study addressing patients with COPD.^30^,^32^ Chan *et al.* showed that higher education levels are positively associated with perceived effectiveness of self-management behaviour among patients with advanced cancer.^33^ Although we did not measure health literacy, it may also play a role in this context. A scoping review of reviews showed that among people with chronic diseases, those with limited health literacy faced barriers primarily related to ‘medical management and the communication and navigation of the healthcare system’.^34^ Concerning the tendency of our relatively high self-management scores in general, people who died between the baseline measurement and the first follow-up time point are not included in the analysis. Furthermore, as Maunsell *et al.* previously proposed, despite advanced stages of cancer, acquired self-management skills might be sustained, or even enlarged.^30^

### Associations of self-management skills with depressive symptoms and quality of life

In our study, higher levels of self-management skills were associated with fewer depressive symptoms and better quality of life. Chan *et al.* also found a significant association between higher levels of perceived effectiveness of self-management behaviour and lower levels of depressive symptoms among people with advanced cancer.^33^ The association between self-management and well-being, and depressive symptoms, in particular, is often described as complex and cyclic. Increases in self-management skills may result in a reduction in depressive symptoms as ‘taking a certain responsibility’ for one’s well-being and having a sense of control over one’s health could help reduce feelings of helplessness.^35^ Vice versa, depressive symptoms could inhibit self-management, presumably because negative emotions may interfere with energy, motivation, or self-efficacy to engage in self-management.^28 36^

### Associations of self-management skills with care utilisation

In this study, we in general did not identify clear significant associations between self-management skills and contact with different types of healthcare disciplines or healthcare organisations, hospital admissions, or emergency care visits. We only found that higher levels of Health services navigation were associated with more nursing care. Furthermore, we found no significant associations between self-management skills and informal care utilisation.

With the pressure on healthcare resources, supporting and enlarging patients’ self-management skills has been put forward in policies to reduce healthcare utilisation and costs.^2 12 37^ It is proposed that patients who are more proficient in taking up care responsibilities themselves and are enabled to ‘function independently in their own home’ have reduced needs for care utilisation such as hospital admissions.^2 37 38^ However, previous studies among patients with chronic conditions also showed that the associations between self-management skills and healthcare utilisation are not always as promising. A meta-analysis showed that among patients with chronic respiratory diseases, the association between self-management skills and hospitalisation days was only marginally significant.^8^ Another systematic review showed that among patients with chronic heart failure, self-management interventions had a positive, although not always significant effect on the number of hospital admissions.^10^ Furthermore, the rationale can go both ways: self-management might also induce healthcare utilisation. First, being aware of one’s symptoms due to self-monitoring could prompt one to seek help.^38^ Additionally, increased empowerment may also imply that individuals are better informed about how to navigate and access healthcare services. This is in line with our association between higher levels of Health service navigation and nursing care. Nurses often serve as the primary point of contact for patients with advanced cancer. Nurses can act as intermediaries between patients and other healthcare providers or empower patients to initiate these connections themselves.

### Clinical implications

Our results add to the body of literature that self-management is associated with better well-being of patients, which is in line with the notion that self-management can benefit patients with advanced cancer.^3^ With the rising number of patients with advanced cancer and higher demands for healthcare resources, there is also a growing interest in the potential benefits of self-management approaches on healthcare utilisation.^2 4^ However, in the present study, we did not find a clear association between self-management skills and healthcare utilisation.

Both patients with advanced cancer and healthcare professionals can face challenges in self-management.^4 15^ Patients’ self-management approaches are highly individual and healthcare professionals also vary in how they provide self-management support.^4 15^ Although the majority of patients with advanced cancer in our study had relatively high levels of self-management skills, healthcare professionals must take into account that deploying self-management skills might be challenging for some individuals with advanced diseases, for example patients with lower levels of educational attainment.^33^ It is proposed that instructive, collaborative, and advisory self-management support roles can all be beneficial and that healthcare professionals who adopt different roles can better meet patients’ diverse needs.^15^

## Conclusions

In a population of patients with diverse types of advanced cancer and relatively high levels of self-management skills, we found a positive association between self-management skills and depressive symptoms and quality of life. This is in line with the notion that self-management can benefit patients with advanced cancer. Skills to navigate the healthcare system were associated with the use of nursing care. However, overall, we found no clear trend indicating that self-management is related to healthcare service and informal care utilisation.

## Data Availability

Data are available on reasonable request.
