# Supplementary material for: Are self-management skills associated with depressive symptoms, quality of life, and healthcare utilisation? A cross-sectional survey among patients with advanced cancer in the Netherlands
Source: BMJ Open. 2026 Feb 27;16(2):e108987. doi: 10.1136/bmjopen-2025-108987 (PMC12958969; doi:10.1136/bmjopen-2025-108987)
Supplement: Supplementary file 1 [file bmjopen-2025-108987.reviewer_comments.pdf]

PEER REVIEW HISTORY

BMJ Open publishes all reviews undertaken for accepted manuscripts. Reviewers are asked to complete a checklist review form and are provided with free text boxes to elaborate on their assessment. These free text comments are reproduced below.

ARTICLE DETAILS

Title (Provisional)

Are self-management skills associated with depressive symptoms, quality of life, and healthcare utilization? A cross-sectional survey among patients with advanced cancer in the Netherlands

Authors

Bakker, Evi M.; van Dongen, Sophie; Witkamp, Erica; Kranenburg, Leonieke W.; van der Rijt, Carin C.D.; Lorig, Kate; Raijmakers, Natasja; van Roij, Janneke; van der Heide, Agnes; Rietjens, Judith

---

VERSION 1 - REVIEW

**Reviewer**	**1**
**Name**	**Sun, Lingyun**
**Affiliation**	**China Academy of Chinese Medical Sciences Xiyuan Hospital**
**Date**	**01-Dec-2025**
**COI**	**None**

---

review for bmjopen-2025-108987

This is a cross-sectional analysis from a cohort study in Netherlands which aimed to evaluate the association between health related self-management and psychological burdens, quality of life and healthcare utilization among cancer population with advanced disease. This topic is clinically relevant and meaningful for patient-centered care in cancer practice. However, there are still some major issues that need to be addressed.

1.In the results part of the abstract, please provide specific scores for all the three scales of the HeiQ questionnaire, because these are main exposures.

2.In the methods part, the author mentioned that the survey was conducted among cancer patients and their relatives. Please clarify whether the survey was completed by patients themselves, or their relatives, or both?

3.Participants were enrolled between 2017 and 2020. However, after the COVID pandemic, people’s health related self-management could change. Please discuss.

4.Whether the “informed consent” is by written or oral?

5.Please provide rationale of choosing these three scales from the HeiQ questionnaire.

6.In the results part, pleas use Arabic numbers instead of words to describe numbers.

7.For HADS and QLQ-C30, the authors used median to describe. However, there was no description on the distribution of these scores to support whether they were normal or not.

8.For all the multivariate analyses, there were no description on what specific background characteristics had been adjusted in both table and main text. In addition, there were no uni-variate analysis on background characteristics and main outcomes-psychological burdens, quality of life and healthcare utilization. By the way, audience might also be interested in the association between background characteristics and main exposures-patient reported self-management.

9.There were multiple main outcomes for the current study, which made the research question not specific and clear enough. For example, psychological burden and health care utilization are two different health care issues. I suggest the authors to narrow down the research question to make the whole manuscript more condensed.

10.Please restructure the discussion part. Usually we don’t discuss the limitations in second paragraph. Also, please discuss the reason why the studied cancer population reported relatively high health related self-management(3 out of 4) which could be a bias due to better health-care system in developed countries.

**Reviewer**	**2**
**Name**	**Yoon, Esther**
**Affiliation**	**Northwestern University Feinberg School of Medicine**
**Date**	**08-Dec-2025**
**COI**	**None**

Great paper. Very much enjoyed reading your discussion section, particularly the clinical implications and understanding the applications of this research to patient care. Some general questions and minor edits below.

Questions:

1. For formal healthcare utilization - was any of the patient's self-report corroborated by medical chart? E.g., pt reported contacting Palliative care provider regarding symptom concern - telephone call or patient portal message found in chart?

2. For contact with healthcare discipline - was this specific to patient-initiated contact only? Or would it also have included routine outreach by medical team for appointment reminders, scheduling, test results, etc?

3. Any sociodemographic data on pre-existing mood disorders, including anxiety and depressive disorders, prior to cancer diagnosis?

4. Any significant sociodemographic differences between patients who had higher vs lower self-management skills?

5. Any association between self-management skills with ANY care utilization, regardless of discipline type?

Minor edits:

1. In results, keep number reporting consistent as all numeric or text-form. E.g., page 7 lines 40-41 ("464 patients", "one thousand one hundred three patients", "four hundred eight-four patients"). E.g., page 8 lines 19-20 ("seventy-nine percent of patients", "16.9% received home care").

2. Results page 12, lines 21-22 ("higher levels of health services navigation were associated with more use of nursing care in the past month"). If a patient could only answer yes/no to having contact with nursing care at least 1x in past month, then I think HSN was associated with having nursing care in past month, not MORE use of nursing care. Unless the study inquired about number of contact with nursing care? If so, then please clarify this in the methods.

3. Discussion: higher self-management in study population might also be influenced by more proficient health literacy (in addition to higher education), which was not assessed as part of study or included in analysis.

4. Discussion page 15 line 34 - not sure if this is just on my end, but the paragraph indent seems 2x larger than usual.

5. Discussion page 16 lines 20-21 ("An example are for example patients with lower levels of educational attainment"). Did you mean to say "For example, patient with lower levels of education attainment"?

VERSION 1 - AUTHOR RESPONSE

<u>Reviewer: 1</u>
Dr. Lingyun Sun, China Academy of Chinese Medical Sciences Xiyuan Hospital
This is a cross-sectional analysis from a cohort study in Netherlands which aimed to evaluate the association between health-related self-management and psychological burdens, quality of life and healthcare utilization among cancer population with advanced disease. This topic is clinically relevant and meaningful for patient-centered care in cancer practice. However, there are still some major issues that need to be addressed.

1. In the results part of the abstract, please provide specific scores for all the three scales of the HeiQ questionnaire, because these are main exposures.
    - All three heiQ subscales are now reported separately.

- Changes in the manuscript – Abstract: *Mean (SD) scores were 3.0 (0.5) for STA, 3.2 (0.4) for SMI, and 3.4 (0.5) for HSN.*

2. In the methods part, the author mentioned that the survey was conducted among cancer patients and their relatives. Please clarify whether the survey was completed by patients themselves, or their relatives, or both?
   - Our cross-sectional questionnaire study is nested in the eQuiPe study, a prospective longitudinal observational cohort study on the experienced quality of care and quality of life of Dutch patients with advanced cancer and their relatives. In our paper, we report on patient data only. To improve clarity, we also included this statement in the methods part of the manuscript.
   - Changes in the manuscript – Methods: *In the current study, we report only self-reported patient data.*

3. Participants were enrolled between 2017 and 2020. However, after the COVID pandemic, people's health related self-management could change. Please discuss.
   - We agree with the reviewer that the COVID-19 pandemic could impact people's health-related self-management. However, patients were enrolled in the eQuiPe study until January 2020. The additional questions regarding self-management skills and depressive symptoms were added at the first follow-up questionnaire: three months after baseline. Data enrollment was thus until March 2020. In March 2020, COVID-19 was recognized as a pandemic in the Netherlands. We therefore believe that the COVID-19 pandemic will likely not have impacted our results, as most questionnaires used in this analysis were completed before the start of the pandemic in the Netherlands.
   - Changes in the manuscript – Methods: *From November 2017 until January 2020 …*

4. Whether the "informed consent" is by written or oral?
   - In the Methods section "Study design and population", we already stated that to participate, patients had to have signed the informed consent form. To improve clarity, in the subsequent Methods section "Study procedure" we now explicitly state that patients needed to provide written informed consent.
   - Changes in the manuscript – Methods: *After giving written informed consent for the eQuiPe study …*

5. Please provide rationale of choosing these three scales from the HeiQ questionnaire.
   - The heiQ questionnaire encompasses eight subscales: 1) Skill and technique acquisition, 2) Self-monitoring and insight, 3) Health service navigation, 4) Positive and active engagement in life, 5) Health directed activities, 6) Constructive attitudes and approaches, 7) Social integration and support, and 8) Emotional distress. We selected Skill and technique acquisition, Self-monitoring and insight, and Health services navigation because we believe they relate most to disease-oriented self-management skills and are therefore most relevant for the purpose of our study. We have now also incorporated this rationale into the manuscript.

- Changes in the manuscript – Methods: *We selected three scales that we considered most related to disease-oriented self-management skills and therefore most relevant for the purpose of our study: Skill and technique acquisition (STA), Self-monitoring and insight (SMI), and Health services navigation (HSN).*

6. In the results part, please use Arabic numbers instead of words to describe numbers.
   - At the BMJ Author Hub, we could not find any guidelines related to handling numerals. However, when it comes to starting a sentence with a number or when the number is less than 10, we believe the general rule is to spell out the number rather than using numerals. As the editor did not make any remarks regarding this topic, we decided to follow this general rule and not alter the manuscript. However, if the editor sees this differently, we are willing to adjust the numerals.

7. For HADS and QLQ-C30, the authors used median to describe. However, there was no description on the distribution of these scores to support whether they were normal or not.
   - In the Methods section "Data analysis" we explain that we applied bootstrapping within the regression analysis to overcome the problem of skewed distributions of the dependent variables. For the description of these distributions, we kindly refer to Table ,1 where the 25–75% percentile scores show that the distributions are somewhat skewed.

8. For all the multivariate analyses, there were no description on what specific background characteristics had been adjusted in both table and main text.
   - We thank the reviewer for this observation. In the Methods section "Data analysis" we explain that the multivariate regression analyses were adjusted for background characteristics that were borderline significantly ($p<0.2$) associated with self-management skills scales in bivariate analyses. To provide full transparency, we also specify in the table legends exactly which background characteristics were included per analysis. We hope this clarification helps to make our analytic approach easier to understand.

9. In addition, there were no uni-variate analysis on background characteristics and main outcomes-psychological burdens, quality of life and healthcare utilization. By the way, audience might also be interested in the association between background characteristics and main exposures- patient reported self-management.
   - Although the reviewer points out interesting univariate analyses, we decided not to include these additional analyses for multiple reasons. First, we believe that the suggested analyses fall outside the scope of our paper. Second, including the proposed univariate analyses will involve many additional analyses, thereby posing a greater risk of Type 1 errors. To strengthen the scientific rigor of our work, we therefore decided not to include additional univariate analyses.

10. There were multiple main outcomes for the current study, which made the research question not specific and clear enough. For example, psychological burden and health care utilization are two different health care issues. I suggest the authors to narrow down the research question to make the whole manuscript more condensed.
    - We are interested in the potential impact of self-management skills on various domains, including both the personal as well as healthcare system levels, as we hypothesize that self-management skills may have an impact on both these domains. We therefore decided to maintain our current research question.

11. Please restructure the discussion part. Usually we don't discuss the limitations in second paragraph.
    - In the BMJ Open Author Guidelines, a recommendation for the overall structure is provided: "a statement of the principal findings; strengths and weaknesses of the study; strengths and weaknesses in relation to other studies, discussing important differences in results; the meaning of the study: possible explanations and implications for clinicians and policymakers; and unanswered questions and future research." We decided to report according to the Author Guidelines and not restructure our Discussion section. However, if the editor sees this differently, we are willing to follow this suggestion and adjust the order.

12. Also, please discuss the reason why the studied cancer population reported relatively high health related self-management (3 out of 4) which could be a bias due to better health-care system in developed countries.
    - We believe that we have found relatively high self-management skill scores because our study population consists of people with high levels of education. We kindly refer to the Discussion section "Levels of self-management skills" in which we elaborate on this hypothesis. We do not think that it is likely that better healthcare systems or policies of developed countries introduce a bias here, as suggested by the reviewer, because in this section, we also currently compare our results with study populations from other developed countries. Therefore, we have decided not to incorporate the reviewer's suggestion.

Reviewer: 2
Dr. Esther Yoon, Northwestern University Feinberg School of Medicine
Comments to the Author: Great paper. Very much enjoyed reading your discussion section, particularly the clinical implications and understanding the applications of this research to patient care. Some general questions and minor edits below.

Questions:

1. For formal healthcare utilization - was any of the patient's self-report corroborated by medical chart? E.g., pt reported contacting Palliative care provider regarding symptom concern - telephone call or patient portal message found in chart?

- Patient's self-reported care utilization was not corroborated by medical charts.

2. For contact with healthcare discipline - was this specific to patient-initiated contact only? Or would it also have included routine outreach by medical team for appointment reminders, scheduling, test results, etc?
   - Contact with healthcare professionals was not limited to patient-initiated contact only. We included a statement in the Methods section "Healthcare service and informal care utilization" to clarify this.
   - Changes in the manuscript – Methods: *Patients were asked whether they had been in any type of contact with various healthcare professionals for their care, and if so, how often.*

3. Any sociodemographic data on pre-existing mood disorders, including anxiety and depressive disorders, prior to cancer diagnosis?
   - Unfortunately, we do not have any information on pre-existing mood disorders prior to the cancer diagnosis. However, one of the inclusion criteria was "no history of severe psychiatric illness", as we currently describe in the Methods section "Study design and population" and Discussion section "Strengths and limitations".

4. Any significant sociodemographic differences between patients who had higher vs lower self-management skills?
   - Those with high education levels had significantly higher scores for the self-management skills self-monitoring and insight compared to patients with low education levels (mean (SD) high education levels: 3.24 (0.44); mean difference with low education levels: 0.18, p=0.001). However, as these additional analyses fall outside the scope of our paper and increase the risk of Type 1 errors, we decided not to include them in the manuscript.

5. Any association between self-management skills with ANY care utilization, regardless of discipline type?
   - Six percent of patients (n = 28) did not have any type of contact with healthcare professionals in the past month. T-tests showed no significant associations with the three subscales of self-management skills. Because of a limited sample size in one of the categories, to ensure scientific rigor, we decided not to include such additional analyses.

<u>Minor edits:</u>

6. In results, keep number reporting consistent as all numeric or text-form. E.g., page 7 lines 40-41 ("464 patients", "one thousand one hundred three patients", "four hundred eight-four patients"). E.g., page 8 lines 19-20 ("seventy-nine percent of patients", "16.9% received home care").
   - We believe the same comment applies here as for the sixth point raised by Reviewer 1: At the BMJ Author Hub, we could not find any guidelines related to handling numerals. However, when it comes to starting a sentence with a number or when the number is less than 10, we believe the general rule is to

spell out the number rather than using numerals. As the editor did not make any remarks regarding this topic, we decided to follow this general rule and not alter the manuscript. However, if the editor sees this differently, we are willing to adjust the numerals.

7. Results page 12, lines 21-22 ("higher levels of health services navigation were associated with more use of nursing care in the past month"). If a patient could only answer yes/no to having contact with nursing care at least 1x in past month, then I think HSN was associated with having nursing care in past month, not MORE use of nursing care. Unless the study inquired about number of contact with nursing care? If so, then please clarify this in the methods.
    - Patients were asked whether they had been in any type of contact with various types of healthcare professionals for their care, and if so, how often. We included a statement in the Methods section "Healthcare service and informal care utilization" to clarify this.
    - Changes in the manuscript – Methods: *Patients were asked whether they had been in any type of contact with various healthcare professionals for their care, and if so, how often.*

8. Discussion: higher self-management in study population might also be influenced by more proficient health literacy (in addition to higher education), which was not assessed as part of study or included in analysis.
    - We included a statement discussing the potential impact of health literacy on self-management.
    - Changes in the manuscript – Discussion: *Although we did not measure health literacy, it may also play a role in this context. A scoping review of reviews showed that among people with chronic diseases, those with limited health literacy faced barriers primarily related to "medical management and the communication and navigation of the health care system" (34).*

9. Discussion page 15 line 34 - not sure if this is just on my end, but the paragraph indent seems 2x larger than usual.
    - We changed the manuscript accordingly.

10. Discussion page 16 lines 20-21 ("An example are for example patients with lower levels of educational attainment"). Did you mean to say "For example, patient with lower levels of education attainment"?
    - We changed the manuscript accordingly.
    - Changes in the manuscript – Discussion: *Although the majority of patients with advanced cancer in our study had relatively high levels of self-management skills, healthcare professionals must take into account that deploying self-management skills might be challenging for some individuals with advanced diseases, for example patients with lower levels of educational attainment (33).*
